# Unstable Points, Ergodicity and Born’s Rule in 2d Bohmian Systems

**DOI:** 10.3390/e25071089

**Published:** 2023-07-20

**Authors:** Athanasios C. Tzemos, George Contopoulos

**Affiliations:** Research Center for Astronomy and Applied Mathematics of the Academy of Athens, Soranou Efessiou 4, GR-11527 Athens, Greece; gcontop@academyofathens.gr

**Keywords:** chaos, Bohmian Quantum Mechanics, Born’s rule

## Abstract

We study the role of unstable points in the Bohmian flow of a 2d system composed of two non-interacting harmonic oscillators. In particular, we study the unstable points in the inertial frame of reference as well as in the frame of reference of the moving nodal points, in cases with 1, 2 and multiple nodal points. Then, we find the contributions of the ordered and chaotic trajectories in the Born distribution, and when the latter is accessible by an initial particle distribution which does not satisfy Born’s rule.

## 1. Introduction

Bohmian Quantum Mechanics [1,2,3,4,5,6,7,8,9] is one of the main interpretations of Quantum Mechanics, where the quantum particles follow deterministic trajectories in spacetime according to the so-called Bohmian equations of motion:

(1) $$\begin{array}{c} m\dot{\vec{r}}=\hbar ℑ\left(\nabla \Psi /\Psi \right), \end{array}$$

where Ψ is a given solution of Schrödinger’s equation (SE) $-\frac{\hbar^{2}}{2m}\nabla^{2}\Psi +V\Psi =i\hbar \frac{\partial \Psi }{dt}$ of Standard Quantum Mechanics (SQM) and *ℑ* stands for the imaginary part. Bohmian equations are first order in time but can be realized as second order in time equations

(2) $$\begin{array}{c} m\ddot{\vec{r}}=-\nabla U, \end{array}$$

when the initial $\dot{\vec{r}}$ is given by Equation (1) and U=V+Q, where *V* is the classical potential and *Q* is the quantum potential [10,11,12,13,14]

(3) $$\begin{array}{c} Q=-\frac{\hbar^{2}}{2m}\frac{\nabla^{2}\left|\Psi \right|}{\left|\Psi \right|}. \end{array}$$

The highly nonlinear character of the Bohmian vector field implies the coexistence (in general) of ordered and chaotic trajectories for a given Bohmian system. Namely, one observes both Bohmian trajectories that are not sensitive to small changes in their initial conditions (ordered trajectories) and Bohmian trajectories with very complex behavior, which changes drastically with a slight change in the initial conditions. Thus, BQM provides us with a natural way to study chaotic phenomena in QM by applying all the techniques of nonlinear dynamics [15].

The study of Bohmian chaos has a history of about 30 years [16,17,18,19,20,21,22,23,24,25,26,27,28]. Most of these works refer to 2d Bohmian systems and only a few of them to 3d systems [19,24,28]. From the first works in the field, it was pointed out that the nodal points of the wavefunction (where Ψ=0) play a major role in the production of chaos since the close encounters between nodal points and Bohmian particles result, in general, in abrupt changes in the directions of the particles [16,19,20]. Moreover, some Bohmian trajectories become trapped around the moving nodal points for finite intervals of time and form a spiral motion around them (Bohmian vortices) [24,25,26].

While Bohmian chaos has been studied in many different quantum systems, as particles in a box [19,20], quantum billiards [20], hydrogen atoms in an electromagnetic field [18], Hénon Heiles potential [17] etc., most results have been found by use of non-interacting quantum harmonic oscillators, where $V=\frac{1}{2}\sum_{i=1}^{N}m_{i}\omega_{i}^{2}x_{i}^{2}$ [16,21,22,23,25,26,27].

Our studies in Bohmian chaos started in 2006 [29] and are focused mainly on the mechanism responsible for the production of chaos in BQM from the standpoint of dynamical systems. In [30,31], we found that whenever a Bohmian particle comes close to a moving nodal point *N*, it gets scattered by an unstable fixed point of the Bohmian flow in the frame of reference $u=x-x_{N},v=y-y_{N}$ centered at the moving nodal point. This is the so-called ‘X-point’, which is defined as the nontrivial solution of the system

(4) $$\begin{array}{c} \frac{du_{X}}{dt}=\frac{dv_{X}}{dt}=0. \end{array}$$

**Figure 1:**
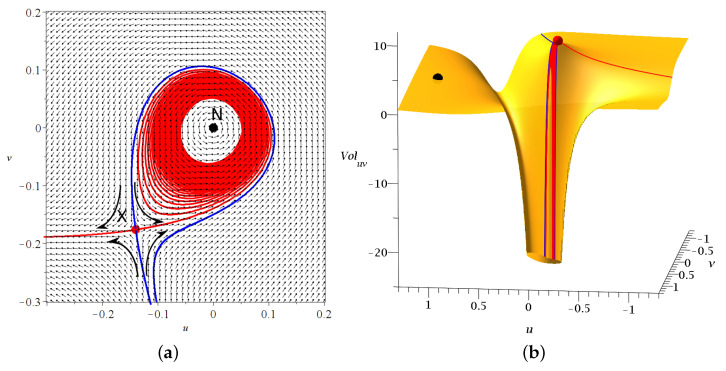
(**a**) A nodal point-X-point complex and the deviations of the trajectories approaching the X-point. (**b**) The total potential close to a nodal point and its corresponding X-point (red dot) and Y-point (black dot). Both figures are drawn in the system (u,v) of the moving nodal point.

The nodal point and its associated nearby X-point form together the ‘nodal point-X-point complex’ (NPXPC), a characteristic geometrical structure of the Bohmian flow close to *N*, which is time dependent due to the non-autonomous nature of the flow (Figure 1a). In fact, the distance between *N* and *X* decreases with the increase of the velocity of *N*, while *N* changes from attractor to repeller from time to time.

From the X-point emanate two stable and two unstable asymptotic curves pointing to opposite directions. In most 2d cases, one asymptotic curve forms a spiral asymptotically approaching the nodal point *N* as t→∞ (Figure 1a) [30].

Trajectories approaching the X-point remain for some time close to its asymptotic curves but then deviate in an irregular way. The result of many such encounters is the emergence of chaos in Bohmian trajectories. On the other hand, the trajectories that remain far from the NPXPCs are ordered. The NPXPC mechanism was first developed for generic 2d systems and then extended to generic 3d systems [32,33]. Moreover, in [34,35], we studied Bohmian order and chaos in 3d harmonic oscillators whose Bohmian trajectories evolve on integral surfaces. These are the so-called ‘partially integrable 3d Bohmian systems’ (see also [28]).

There are wavefunctions with 0,1,2 up to infinitely many nodal points. In general, the larger the number of nodes (i.e., of the NPXPCs), the stronger the appearance of chaos. However, even in the case of infinitely many nodal points, one still finds regions of order in the configuration space [36]. Moreover, the number of nodal points may change from time to time. For example, two nodal points may join and disappear for a time interval [31]. There are also cases where the nodal points are fixed (they do not move), as in [28], and cases with both fixed and moving nodal points [37].

In [38], we found that while *Q* and *V* go to −∞ at *N*, the X-point is very close to the local positive maximum of *Q* and *V*. This maximum increases with the velocity of the nodal point (Figure 1b).

Since BQM is a trajectory-based quantum theory, a solid understanding of the production of chaos in Bohmian trajectories is necessary for each of its aspects. In the past, we applied the NPXPC mechanism in order to understand the interplay of ordered and chaotic trajectories in various particle distributions and tested when it allows (or not) the dynamical approach of Born’s Rule (BR) distribution $P={\left|\Psi \right|}^{2}$ when $P_{0}\neq {\left|\Psi_{0}\right|}^{2}$. In fact, BR (according to which the probability density of finding a particle in a certain region of space is $P={\left|\Psi \right|}^{2}$), is not an axiom in BQM as in SQM. We can, in principle, consider a particle distribution with $P_{0}\neq {\left|\Psi_{0}\right|}^{2}$. And it is well known that if BR is initially (at t=0) satisfied, i.e., if $P_{0}={\left|\Psi_{0}\right|}^{2}$, then it is satisfied also at all subsequent times. On the other hand, if $P_{0}\neq {\left|\Psi_{0}\right|}^{2}$, then as t→∞, the distribution *P* tends, in general, to satisfy Born’s rule. The origin of BR in BQM has been studied by many authors in the past [39,40,41,42,43,44,45].

In our studies of the Bohmian trajectories of entangled qubit systems of the form

(5) $$\begin{array}{c} \Psi =c_{1}Y_{R}\left(x\right)Y_{L}\left(y\right)+c_{2}Y_{L}\left(x\right)Y_{R}\left(y\right), \end{array}$$

where $Y_{R}$ (right) and $Y_{L}$ (left) are coherent states of the quantum harmonic oscillators given by Equations (5)–(9) of [46] and $c_{1},c_{2}$ weights with $|c_{1}{|}^{2}+{\left|c_{2}\right|}^{2}=1$, we found that the chaotic trajectories are ergodic. Namely, they share a common long-time distribution of the points of the trajectories in the configuration space. However, the ordered trajectories are not ergodic and their distribution of points is limited in the configuration space. Thus, unless the initial distribution has the correct proportion and distribution of ordered trajectories, the final distribution as t→∞ does not reach the Born distribution. Of course, there are particular cases with no ordered trajectories, such as the maximally entangled case ($c_{1}=c_{2}=\sqrt{2}/2$), where any initial distribution of trajectories tends to satisfy Born’s rule after a long time. On the other hand, we have the product states with $c_{1}=0$ or $c_{2}=0$, where all trajectories are ordered and form Lissajous figures (see also [47]).

Ergodicity was found to be very useful since we can gain complete information about the long-time behavior of chaotic trajectories by integrating only a few of them. However, there are cases that are not globally ergodic. In the next section, we present a particular case of this phenomenon, where the ergodic pattern of chaotic trajectories has only a local character, i.e., there are particular sets of trajectories with the same pattern.

In the present paper, we find that a contribution to chaos and ergodicity (global or local) is provided also when a trajectory approaches an unstable point of the Bohmian flow in the inertial frame of reference (x,y). We call these unstable points ‘Y-points’ and find them in wavefunctions with 1, 2, and multiple nodes. Finally, we study—in all of these cases—the chaotic and ordered trajectories inside the Born distribution and calculate the corresponding ergodic patterns, commenting on the accessibility of Born’s distribution by arbitrary initial distributions.

The structure of the paper is the following: In Section 2, we study the Y-points of a wavefunction of a single nodal point to observe local ergodicity. We then discuss the ratio between ordered and chaotic trajectories inside the Born distribution. In Section 3, we do the same in the case of 2 nodal points, and in Section 4, we present an example of a complex multinodal wavefunction. In Section 5, we draw our conclusions. Finally, in Appendix A, we describe a practical method for the discrimination between ordered and chaotic trajectories based on the existence of ergodicity (local or global).

## 2. One Nodal Point

A case with one nodal point is given by the wavefunction:

(6) $$\begin{array}{c} \Psi =\Psi_{0,0}+a\Psi_{1,0}+b\Psi_{1,1} \end{array}$$

where

(7) $$\begin{array}{cc} & \Psi_{0,0}=e^{-i\left(1+c\right)t/2}e^{-1/2\;cy^{2}-1/2\;x^{2}}, \end{array}$$

(8) $$\begin{array}{cc} & \Psi_{1,0}=e^{-i\left(3+c\right)t/2}e^{-1/2\;cy^{2}-1/2\;x^{2}}x, \end{array}$$

(9) $$\begin{array}{cc} & \Psi_{1,1}=e^{-3\;i\left(1+c\right)t/2}e^{-1/2\;cy^{2}-1/2\;x^{2}}x\sqrt{c}y. \end{array}$$

$\Psi_{n1,n2}$ stands for a 2d stationary state [21] with quantum numbers $n_{1}$ in *x* and $n_{2}$ in *y*. This case was considered in [16,30] with a=b=1 and $c=\sqrt{2}/2$ and corresponds to the classical potential $V=(x^{2}+c^{2}y^{2})/2$. This wavefunction belongs to the simplest form of 2d wavefunctions, which can exhibit chaos as already shown in [22]. The nodal point is at

(10) $$\begin{array}{c} x_{N}=-\frac{\sin[(c+1)t]}{a\sin(ct)},\;y_{N}=-\frac{a\sin(t)}{b\sqrt{c}\sin\left[\left(c+1\right)t\right]}. \end{array}$$

The Bohmian equations of motion in this case read:

(11) $$\begin{array}{cc} & \dot{x}=-\frac{a\sin\left(t\right)+b\sqrt{c}y\sin\left(ct+t\right)}{G} \end{array}$$

(12) $$\begin{array}{cc} & \dot{y}=-\frac{b\sqrt{c}x\left(ax\sin\left(ct\right)+\sin\left(ct+t\right)\right)}{G}, \end{array}$$

where $G=1+2\;ax\cos\left(t\right)+2\;b\sqrt{c}xy\cos\left(ct+t\right)+a^{2}x^{2}+2\;ab\sqrt{c}x^{2}y\cos\left(ct\right)+b^{2}cx^{2}y^{2}$. Thus, there is also an unstable point (Y-point) on the plane x−y

(13) $$\begin{array}{c} x_{Y}=0,\;y_{Y}=-\frac{a\sin(t)}{b\sqrt{c}\sin\left[\left(c+1\right)t\right]}, \end{array}$$

where $\dot{x}=\dot{y}=0$, in the sense that in the inertial frame of reference (x,y), this is an unstable fixed point at a given time *t*. We note that *Y* is always on the axis x=0 and with the same *y* as the nodal point, i.e., $y_{Y}=y_{N}$.

If we linearize the Bohmian field around the Y-point, we find that

(14) $$\begin{array}{cc} & \dot{\xi }=-b\sqrt{c}\sin\left[\left(c+1\right)t\right]\eta \end{array}$$

(15) $$\begin{array}{cc} & \dot{\eta }=-b\sqrt{c}\sin\left[\left(c+1\right)t\right]\xi , \end{array}$$

where $\xi =x-x_{Y}$ and $\eta =y-y_{Y}$. Thus, the Jacobian matrix of the Bohmian flow $\dot{\vec{r}}=F$ at the Y-point $J_{i,j}={\left(\frac{\partial F_{i}}{\partial x_{j}}\right)}_{Y}$ is symmetric, with $J_{1,1}=J_{2,2}=0$ and $J_{1,2}=J_{2,1}=-b\sqrt{c}\sin\left[\left(c+1\right)t\right]$. The corresponding eigenvalues are $\lambda_{1,2}=\pm b\sqrt{c}\sin\left[\left(c+1\right)t\right]$. Since they are real and of opposite sign, the Y-point is a hyperbolic fixed point of the Bohmian flow [48]. Thus, if we fix time *t* in the right-hand side of the Bohmian equations and integrate the corresponding autonomous flow in a new time *s* (see [31]), then we can draw the unstable/stable invariant curves of the Y-point (integrated in positive/negative time *s*), similarly to the case of the X-point (see Figure 1).

Moreover, from the linearized system, we find:

(16) $$\begin{array}{c} \frac{d\xi }{d\eta }=\frac{\eta }{\xi }\;\Rightarrow \;\xi^{2}-\eta^{2}=C. \end{array}$$

Therefore, the trajectories close to *Y* are approximately hyperbolas. If C>0, the trajectories on the left of the unstable point *Y* (see the flow close to η=0), as they approach *Y*, deviate initially to the left, and the trajectories on the right of *Y* deviate initially to the right. These trajectories are chaotic. On the other hand, if C<0, the trajectories above the Y-point move to the left, and the trajectories below the Y-point move initially to the right. These trajectories are ordered.

**Figure 2:**
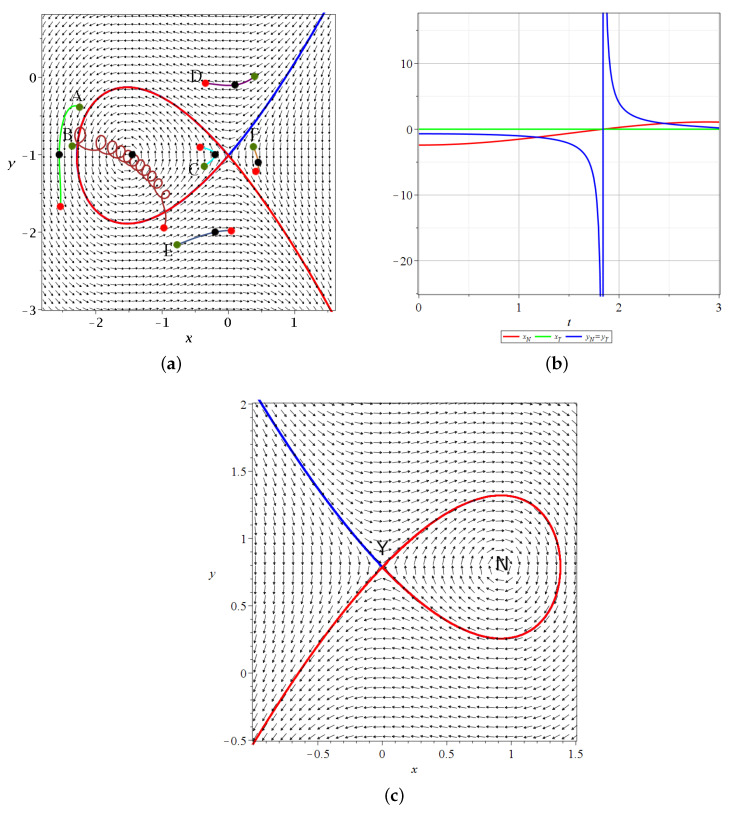
(**a**) A snapshot of the time-dependent Bohmian flow and the invariant curves of the Y-point at t=1 along with various Bohmian trajectories integrated from t=0 (green dots) up to t=3 (red dots) in the case of Equation (6) ($a=b=1,c=\sqrt{2}/2$). The black dots correspond to t=1, i.e., to the flow snapshot. We note that the flow changes in t∈[0,3], but we still understand the form of the trajectories by reading the coordinates of the nodal point ($x_{N}$ red) and the Y-point ($x_{Y}$ green and $y_{N}=y_{Y}$ blue) for t∈[0,3], as shown in (**b**). There, we see that $y_{N}=y_{Y}$ passes from −∞ to *∞* (at t≃1.84), $x_{N}$ changes its sign from negative to positive. (**c**) The Bohmian flow and the invariant curves of the Y-point at t=2.5, where $x_{N}>0$. The stable/unstable invariant curves are calculated in positive/negative time *s*.

In Figure 2a, we show the invariant curves of *Y* (stable (blue) and unstable (red)) at t=1. Close to *Y*, these curves are η=±ξ.

We note that in Figure 2a, the nodal point is on the left of the Y-point. However, while the Y-point is always on the x=0 axis ($x_{Y}=0$), the nodal point moves from negative *x* to positive *x* passing through x=0 at t=π/(1+c)≃1.84. In fact, for t=0, we have ($x_{N}=-2.4,y_{N}=-0.70$). For t=1, we have $(x_{N}=-1.53,y_{N}=-1.01$ and for t=1.5, we have ($x_{N}=-0.63,y_{N}=-2.16)$. For a little larger *t*, (t=1.84) the value of $y_{N}=y_{Y}$ goes to −∞ and $x_{N}=0$. For larger *t*, $x_{N}>0$.

The value of $y_{N}=y_{Y}$ starts at y<0 for t=0 and decreases to −∞ for t=1.84 (see Figure 2b). Then, it jumps to *∞* and decreases to −∞ at t=2π/(c+1)≃3.68. In Figure 2c, we show the invariant curves of the Y-point at t=2.5 where the nodal point lies on the right of the y-axis. The flow around *Y* is then quite different (the reverse) from that of Figure 2a.

In Figure 2a, we also have a number of trajectories of quantum particles from t=0 (green dots) to t=1 (black dots) and t=1.5 (red dots). Trajectory A on the left goes downward. It remains far from the nodal point (at least for this interval of time). Trajectory B approaches the nodal point *N* and forms loops around it following the motion of *N* (Bohmian vortex) downward and to the right (see in Figure 2b the coordinates of $x_{N},y_{N}$ as functions of time). However, at a little larger *t*, the nodal point moves fast downward and trajectory B no longer follows *N*, as *t* goes to t=1.5 and beyond it. We note that the smaller the distance between the trajectory and the moving node *N*, the faster the spiral motion of the particle around *N*. Thus, for a reliable calculation of the trajectory, we use a high accuracy adaptive numerical integration method (see Appendix A). Trajectory *C* approaches the Y-point from t=0 to t=1 and then is deflected to the left. Trajectory *D* moves from the left to the right crossing the x=0, axis up to a small positive *x*, but later it turns to the left because the Y-point has moved downward quite a lot, so that for t=1.5, it is well below the trajectory E.

Finally, the trajectory F moves initially a little to the right (from t=0 to t=1), but later, as the Y-point moves fast downward, it is above the position of the blue asymptotic curve for t=1.5 and thus it is directed to the left.

Whenever a trajectory comes close to a nodal point *N*, it gets scattered by a nearby unstable point (X-point) in the frame of reference of *N* [30,31]. The same happens with the Y-points that are unstable points in the inertial (x,y) frame of reference. These scattering events are responsible for the emergence of chaos in BQM. They can be monitored by the so-called ‘stretching number’ [49] that is strongly related to the Lyapunov characteristic number (LCN), which identifies order and chaos in dynamical systems [48].

In particular, if we consider two nearby trajectories at $t=t_{0}$ and their deviation vector $\xi_{k}$ at $t=kt_{0},k=1,2,\ldots$ then the ‘finite time Lyapunov characteristic number’ is defined as

(17) $$\chi =\frac{1}{kt_{0}}\sum_{a=1}^{k}a_{i},$$

where

(18) $$a_{k}=\ln\left(\frac{\xi_{k+1}}{\xi_{k}}\right)$$

is the ‘stretching number’. The LCN is the limit of χ at k→∞. LCN is zero for ordered trajectories and positive for chaotic trajectories.

**Figure 3:**
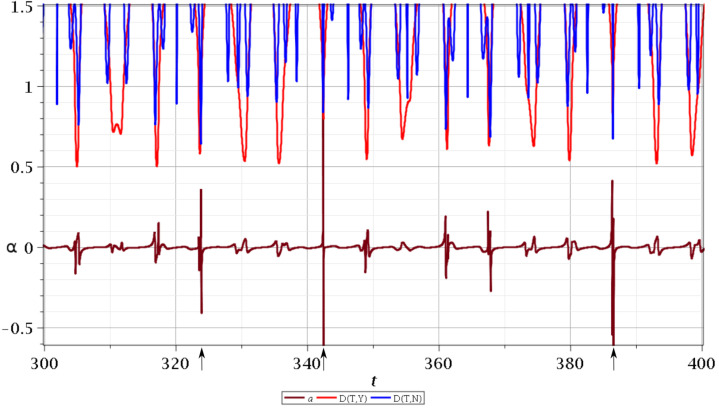
The distance between chaotic Bohmian trajectory and the nodal point (blue curve of the upper part) and the Y-point (red curve of the upper part) and the corresponding stretching number *a* for t∈[300,400]. We observe that most of the significant scattering events correspond to the close approaches to the nodal points (and their associated X-points).

Whenever a Bohmian trajectory approaches an unstable point, the stretching number undergoes a shift. This is shown in Figure 3 where we plot in blue color the distance of a chaotic Bohmian trajectory from the nodal point (which is close to its associated X-point) and in red color the corresponding distance from the Y-point. We see that, in principle, all the shifts of the stretching number *a* correspond to the local minima of blue and red curves, implying that these are the moments where chaos is produced.

The three large shifts (marked with arrows) are produced when the distance between the trajectory and the nodal point (and its corresponding X-point) is very small. All the smaller shifts that could not be explained by close approaches to an X-point can now be explained as scattering events between the trajectory and the Y-point.

**Figure 4:**
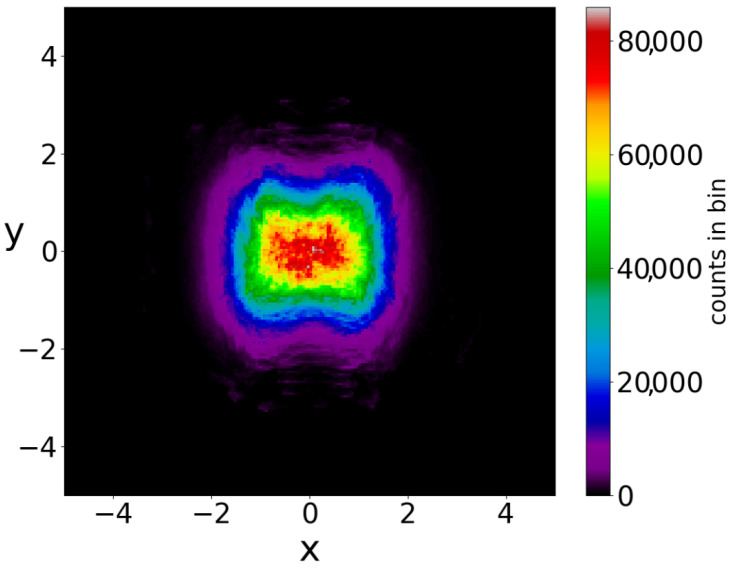
The distribution of the points (at every Δt=0.05) of 3000 trajectories when the initial distribution satisfies BR, up to t=3000.

If we consider a square grid inside the configuration space and count how many times a trajectory (or multiple trajectories in the case of a distribution of particles) has crossed every cell of the grid up to a given time, we can draw ‘colorplots’ like that of Figure 4 (for more details, see [50]). These colorplots acquire a certain pattern after long times.

**Figure 5:**
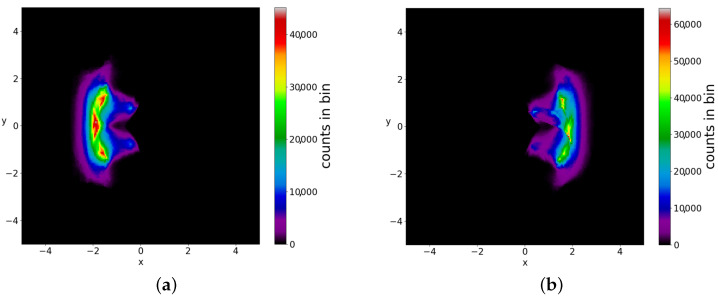
The colorplots of two locally ergodic–chaotic trajectories separately up to $t=2\times 10^{6}$ (**a**) on the left and (**b**) on the right of *y*-axis in the single node case.

The chaotic trajectories of this wavefunction are locally ergodic (due to the Y-point), in the sense that the long-time colorplots of two nearby chaotic trajectories with x(0)<0 or x(0)>0 are the same (Figure 5a,b). However, as the colorplots of the trajectories with x(0)<0 and x(0)>0 are not the same, these cannot be regarded as (globally) ergodic trajectories. In fact it is probable that every chaotic trajectory is locally ergodic, since the points of any given chaotic trajectory fill a certain area on the x−y plane. Then, chaotic trajectories starting in the same area, probably fill this area.

**Figure 6:**
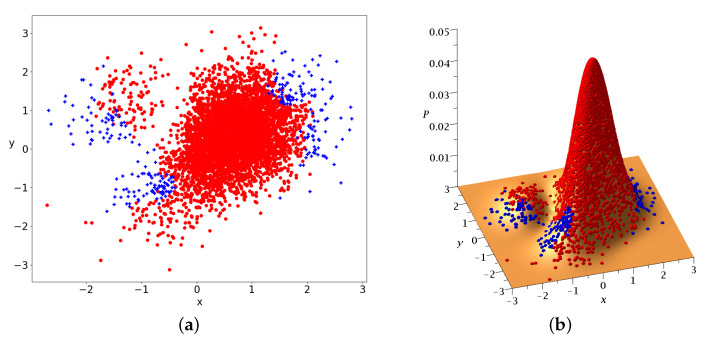
5000 initial conditions, in the case of a single nodal point, distributed according to BR at t=0 (**a**) on the x−y plane and (**b**) projected on $P_{0}={\left|\Psi_{0}\right|}^{2}$. Blue/red initial conditions produce chaotic/ordered Bohmian trajectories.

**Figure 7:**
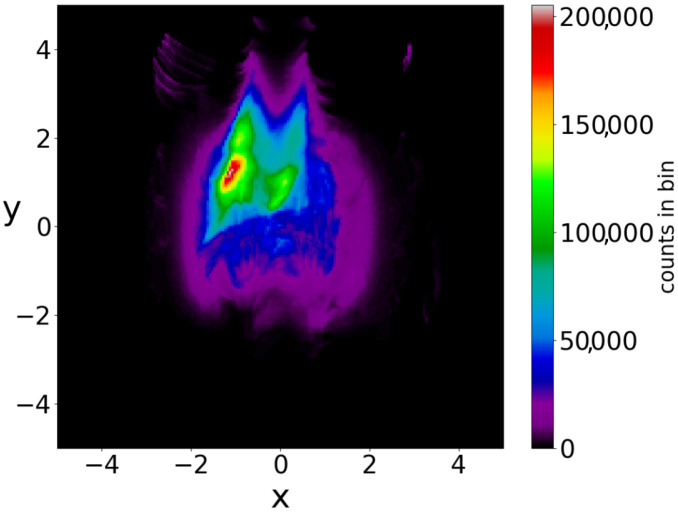
Colorplot of 5000 trajectories (in the case of a single nodal point) with $P_{0}\neq {\left|\Psi_{0}\right|}^{2}$, at t=3000. It is very different from that of the BR distribution in Figure 4.

In a Born distribution of particles, the proportion of ordered trajectories is approximately 95% and they cover the central part of the x−y plane, while the proportion of chaotic trajectories is only 5% (Figure 6a,b) (for the detection of order and chaos see the Appendix A). However, the ordered trajectories are not ergodic. As a consequence, if the initial distribution is different from that of Born’s rule, then the long-term distribution of the points of these trajectories (colorplot) does not approach the BR colorplot. An example is shown in Figure 7, where we see the limiting colorplot from a particular initial distribution different from the Born distribution. This colorplot is quite different from that of Figure 4. Therefore, the trajectories with $P_{0}\neq {\left|\Psi_{0}\right|}^{2}$ do not reach, in general, BR in the long run.

## 3. Two Nodal Points

If the wavefunction, Ψ contains terms $\Psi_{n1,n2}$ with $n_{1}$ and/or $n_{2}$ larger than 1, we have more than 1 nodal point (for certain intervals of values of *t*). A simple case with two nodal points is that of the wavefunction

(19) $$\begin{array}{cc} & \Psi =\Psi_{0,0}+a\Psi_{2,0}+b\Psi_{1,1}, \end{array}$$

where

(20) $$\begin{array}{cc} & \Psi_{2,0}=e^{-i/2\left(5+c\right)t}e^{-1/2\;cy^{2}-1/2\;x^{2}}\left(x^{2}-1\right). \end{array}$$

In this case, the nodal points satisfy the equations

(21) $$\begin{array}{cc} & a\cos\left(2t\right)\left(x^{2}-1\right)+b\sqrt{c}\cos\left[\left(c+1\right)t\right]xy=0, \end{array}$$

(22) $$\begin{array}{cc} & a\sin\left(2t\right)\left(x^{2}-1\right)+b\sqrt{c}\sin\left[\left(c+1\right)t\right]xy=0 \end{array}$$

and its solutions are

(23) $$\begin{array}{cc} & x_{N}=\pm {\left[1-\frac{sin(c+1)t}{a\sin[(c-1)t]}\right]}^{1/2}, \end{array}$$

(24) $$\begin{array}{cc} & y_{N}=\frac{\sin(2t)}{b\sqrt{c}x_{N}^{\prime }\sin\left[\left(c-1\right)t\right]}. \end{array}$$

The two nodal points are symmetric with respect to the origin. When

(25) $$\begin{array}{c} \left|\frac{\sin[(c+1)t]}{a\sin[(c-1)t]}\right|\geq 1, \end{array}$$

the two points join at the origin and disappear for a certain interval of time. On the other hand, the unstable points have $\dot{x_{Y}}=\dot{y_{Y}}=0$, i.e., are solutions of the equations

(26) $$\begin{array}{cc} & \left[b\sqrt{c}\left(a\sin\left[\left(c-1\right)t\right]\left(x^{2}+1\right)-\sin\left[\left(c+1\right)t\right]\right)y-2a\sin\left(2t\right)x\right]/G=0, \end{array}$$

(27) $$\begin{array}{cc} & \left[\left(a\sin\left[\left(c-1\right)t\right]\left(x^{2}-1\right)+\sin\left[\left(c+1\right)t\right]\right)x\right]/G=0, \end{array}$$

with

(28) $$\begin{array}{cc} G= & 2\sqrt{c}abx^{3}y\cos\left(ct-t\right)+cb^{2}x^{2}y^{2}-2\sqrt{c}abxy\cos\left(ct-t\right)+a^{2}x^{4}+2\sqrt{c}bxy\cos\left(ct+t\right)\\ & +2ax^{2}\cos\left(2t\right)-2a^{2}x^{2}-2a\cos\left(2t\right)+a^{2}+1. \end{array}$$

**Figure 8:**
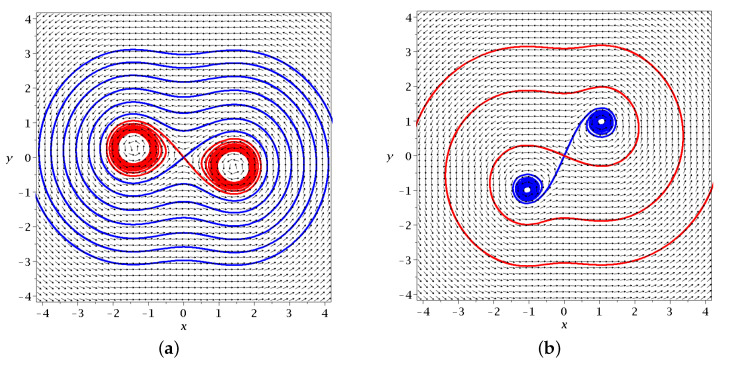
The stable (blue) and unstable (red) asymptotic curves of the Y-point in the case of two nodal points for (**a**) t = 1.5 and (**b**) t = 1.8 in the case of Equation (19) ($a=1.23,b=1,c=\sqrt{2}/2$). We observe the change in the behavior of the nodal points from attractors to repellers.

Thus, there is only one Y-point, which is always at the origin (0,0). Its asymptotic curves are of the form of Figure 8.

**Figure 9:**
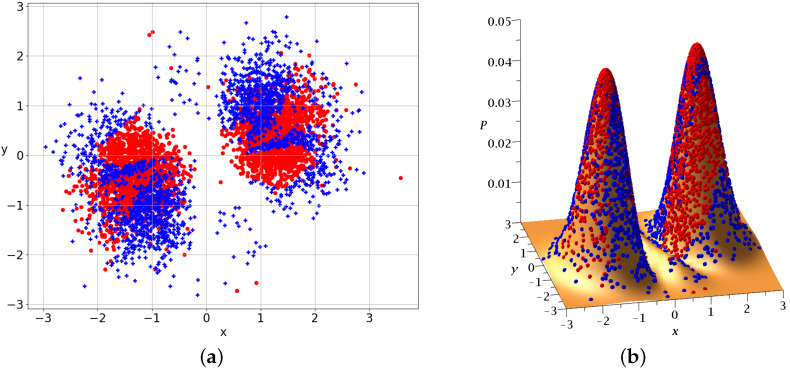
5000 initial conditions in the case of 2 nodal points distributed according to BR at t=0 (**a**) on the x−y plane and (**b**) projected on $P_{0}={\left|\Psi_{0}\right|}^{2}$. Blue/red initial conditions produce chaotic/ordered Bohmian trajectories.

In the case of Equation (19), we have both chaotic and ordered trajectories. As an example, we consider $a=1.23,b=1,c=\sqrt{2}/2$. The initial Born distribution of 5000 particles is represented in Figure 9a,b. The blue crosses represent chaotic trajectories and the red dots represent ordered trajectories. We observe two concentrations of points approximately symmetric with respect to the origin (0,0).

The Born distribution contains approximately 42% chaotic trajectories and 58% ordered trajectories. Therefore, the ordered trajectories play a major role in this case.

**Figure 10:**
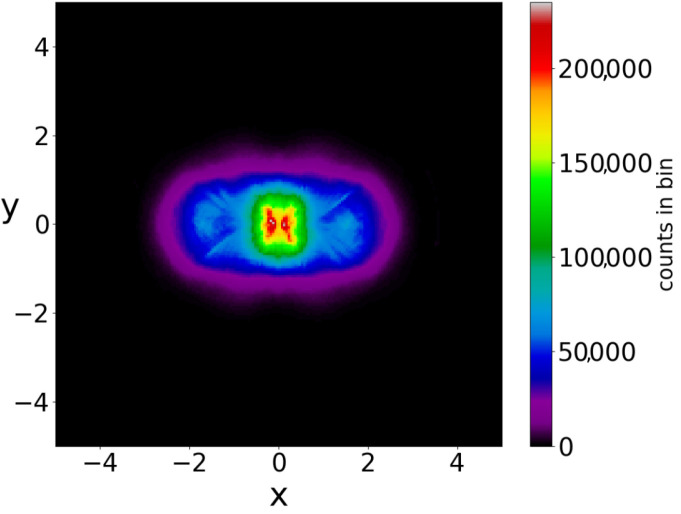
The colorplot of the points of 5000 trajectories (in the case of two nodal points) initially satisfying BR, up to t=3000.

**Figure 11:**
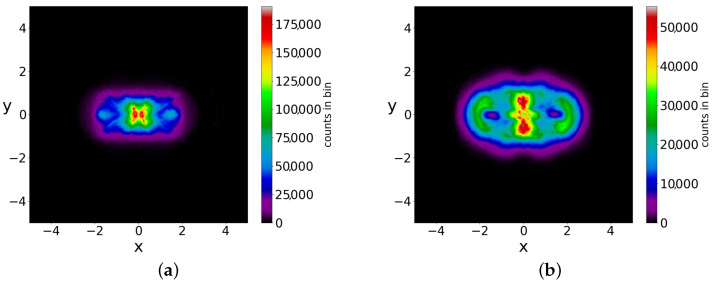
The colorplots of (**a**) the ordered and (**b**) of the chaotic trajectories in an initial BR distribution (in the case of two nodal points).

If we take the distribution of the points of the 5000 trajectories up to t=3000 (points at every Δt=0.05) we find the colorplot of Figure 10. This is approximately symmetric with respect to the axis x=0 and with two maxima, one on the left and one on the right of the origin. However, if we plot separately the points of the ordered and of the chaotic trajectories, then we find two very different distributions (Figure 11a,b). In Figure 11a (2880 ordered trajectories), the colorplot is similar to the total colorplot (Figure 10), while the colorplot of Figure 11b (2120 chaotic trajectories) is quite different. This fact shows that the total colorplot is dominated by the ordered trajectories.

**Figure 12:**
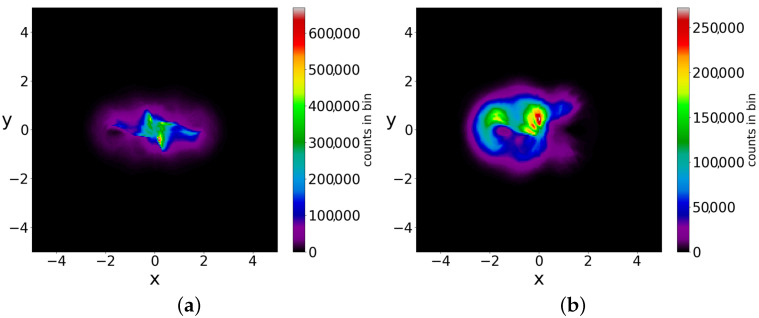
The colorplots of 5000 trajectories in two initial distributions with $P_{0}\neq {\left|\Psi_{0}\right|}^{2}$, up to t=3000. The shape of (**a**) is different from that of (**b**) and they both are very different from that of the BR distribution (Figure 10).

The chaotic trajectories in this case are ergodic (while the ordered trajectories are, of course, not ergodic). Therefore, if we take an initial distribution different from BR, we do not reach the Born rule distribution after a long time. In fact, in the two examples of Figure 12a,b, where we have $P_{0}\neq {\left|\Psi_{0}\right|}^{2}$, the final colorplot is different from the colorplot of Figure 10, which corresponds to BR.

## 4. Multiple Nodal Points

**Figure 13:**
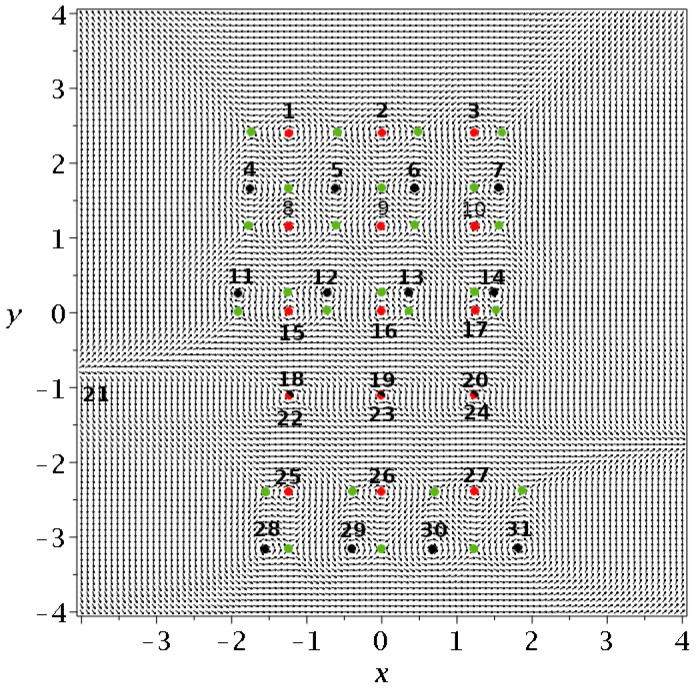
The Bohmian flow along with the stationary nodal points (red dots), the moving nodal points (black dots) and the Y-points (green dots) at t=0.1.

We consider the critical points of a system with multiple nodal points that was studied in [37]. This refers to the wavefunction

(29) $$\begin{array}{c} \Psi =\Psi_{3,3}+\Psi_{3,4}+\Psi_{4,5}, \end{array}$$

with $\omega_{1}=1$ and $\omega_{2}=\sqrt{2}/2$ (Figure 13). In this case, there are 31 nodal points of two types. First, there are five triplets consisting of three fixed nodal points each, that have the same *y* and do not change in time. Then, there are four quadruples of moving nodal points, again with the same *y*. One of the four nodal points of every quadruple moves to infinity in *x* and returns. The moving nodal points move upwards in time and from time to time, three of each set collide with the fixed nodal points and continue upward. They reach +∞ in *y* and later they appear upward from y=−∞.

The Y-points that we consider in the present paper are unstable points that are shown as green points in Figure 13 for a time t=0.1. These are again, of two types. First, we have four Y-points of the same *y* of a triplet of fixed nodal points and three Y-points with the same *y* as every set of moving nodal points. The Y-points move only horizontally in the first case while they move upward together with the moving *N* points (they share the same *y* at every time).

**Figure 14:**
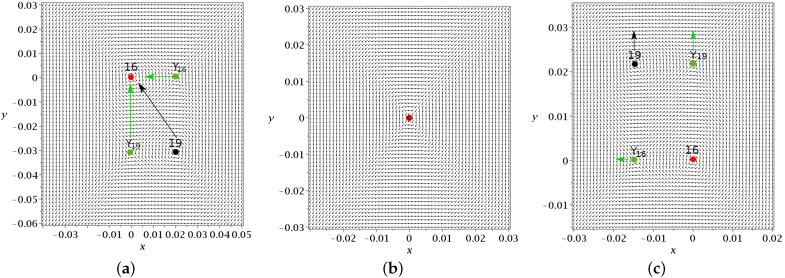
Details of the collision between the moving nodal point 19 and the fixed nodal point 19 at y=0. (**a**) Before the collision (t=1.8), the nodal point 19 and a nearby green point $Y_{19}$ move toward the fixed point 16 together with the green point $Y_{19}$ on the left of 19 (see the arrows). (**b**) At $t=t_{col}=1.8403$ we observe the collision. (**c**) After the collision (t=1.87), the moving nodal point 19 and $Y_{19}$ are above point 16 but $Y_{19}$ is now on the right of the nodal point 19 and the green point $Y_{16}$ has moved to the left of 16.

When three moving nodal points collide with three fixed nodal points, the three green points of the level of the quadruple and three out of four green points of the level of the triplet also collide at the same three points. The fourth green point then goes to infinity. The details are shown in Figure 14a (before the collision) and Figure 14c (after the collision).

**Figure 15:**
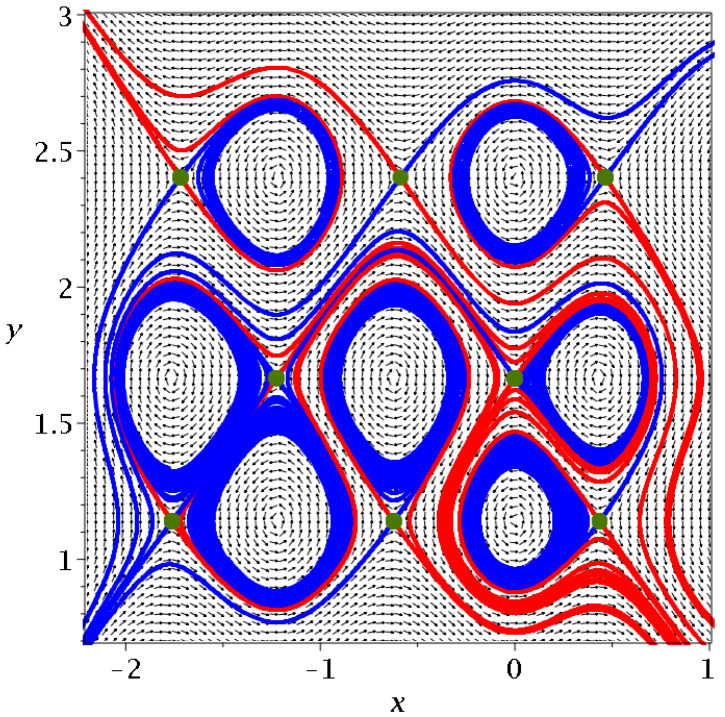
The asymptotic curves of some Y-points of the upper left corner of Figure 13, stable (blue) and unstable (red).

Every Y-point has its asymptotic curves (stable blue and unstable red). An example is shown in Figure 15. Some asymptotic curves surround more than one fixed point. On the other hand, some asymptotic curves, both blue and red, extend to infinity. Many curves are, in fact, double. For example, the red curve that goes up to the upper left corner of Figure 15 contains one red curve from the top left green point and another red curve that starts from this green point downward to the right, surrounds the nodal point on the right of the green point and then comes close to the green point again but a little above it and continues as a close parallel of the first red curve, upward and to the left. Above this double red curve, there is a single red curve from the upper middle green point of Figure 15 to the left that goes to y=∞. The other red curve from this green point goes downward and, after some oscillations, it goes to x=∞.

**Figure 16:**
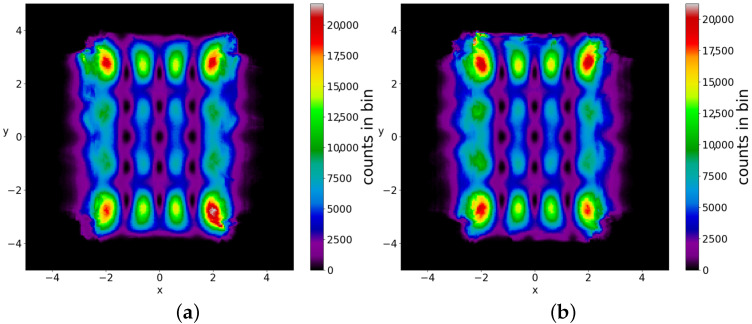
Colorplots of two different chaotic trajectories in the case of multiple nodal points up to $t=5\times 10^{6}$: (**a**) x(0)=2.8,y(0)=0.8 and (**b**) x(0)=−3,y(0)=−1. They are very similar, i.e., they are approximately ergodic.

**Figure 17:**
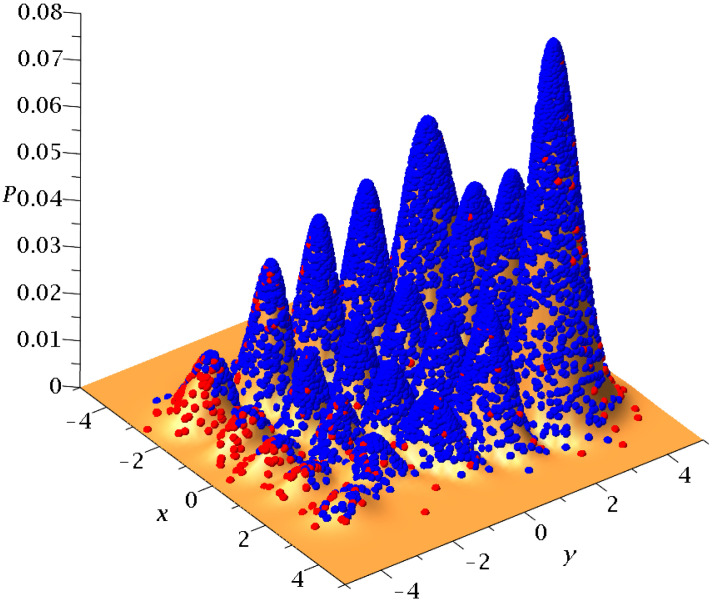
10,000 initial conditions in the case of multiple nodal points distributed according to Born’s distribution at t=0, chaotic (blue) and ordered (red).

The points of the chaotic trajectories fill more or less densely a square of dimension (−3<x<3, −3<y<3) with maxima near the Y-points (the four maxima near the top and bottom are stronger, while the maxima at y=0 are much weaker). Moreover, two different initial conditions give approximately the same color plots (Figure 16a,b), i.e., the chaotic trajectories are ergodic. In a Born initial distribution, there are 95% chaotic trajectories and only 5% ordered trajectories (Figure 17). Therefore, in this case, BR is almost always approximately accessible. In general, if there is a large number of scattered nodal points in the configuration space then BR is accessible. We note however, that even if we have an infinity of nodal points (as in the case described in ([36]), Born’s rule is not, in general, accessible when there is a large number of ordered trajectories.

## 5. Conclusions

In this paper, we presented several characteristics of the Bohmian dynamics in a 2d harmonic oscillator, in the cases of 1, 2, and multiple nodal points.

In particular, we focused on the unstable points of the Bohmian flow in the inertial frame of reference (x,y), which also contribute to the chaotic dynamics besides the unstable points in the frame of reference of the nodal points, the X-points. The Y-points are not as significant as the X-points in the production of chaos in the case of a single nodal point, but they are responsible for the phenomenon of local ergodicity. Namely, in this case, they do not allow the chaotic trajectories to pass through the x=0 axis, and thus, their long-time distribution of points is the same only for the trajectories in the part of the configuration space with $x_{0}>0$ or with $x_{0}<0$.

In the case of the 2 nodal points, the ergodic behavior of the chaotic trajectories is global. However, there are a lot of ordered trajectories (which are obviously not ergodic) in the Born distribution, that prevent the dynamical approach of Born’s rule in many initial distributions with $P_{0}\neq |\Psi_{0}^{2}$. Only if the ratio between ordered and chaotic trajectories is the same as that of the BR distribution (and the ordered trajectories are BR distributed) do we finally reach BR in the long run. And in the special case of the single nodal point, we have to take also into account the proportion between the left and right locally ergodic chaotic trajectories in the BR distribution.

In the case of multiple nodal points, there are plenty of X-points and Y-points in the configuration space that scatter the incoming trajectories and thus produce chaos and global ergodicity. In fact, the great majority of trajectories in Born’s distribution are chaotic (95%); therefore, the role of the ordered trajectories is very small. Thus, BR is accessible to a much larger number of initial distributions than in the few nodal points cases.

In all cases, we presented the local form of Bohmian flow in the close neighborhood of the Y-points by plotting the invariant curves emanating from them, i.e., the routes of chaos. Moreover, we managed to discriminate the ordered and chaotic trajectories inside large ensembles of initial conditions exploiting the ergodic (local or global) character of chaotic trajectories. In fact, ergodicity is useful since the form of the common limiting distribution (colorplot) of ergodic trajectories can be used in practical algorithms for quick and reliable discrimination between ordered and chaotic trajectories, as shown in Appendix A.

Further work needs to be conducted in complex systems with a larger number of nodal points and with several geometries in the configuration space.

Finally, we want to emphasize the fact that an initial distribution of Bohmian particles with $P_{0}={\left|\Psi_{0}\right|}^{2}$ contains, in general, both ordered and chaotic trajectories. Therefore, as we noticed in the introduction, if $P_{0}\neq {\left|\Psi_{0}\right|}^{2}$ then we can reach BR in the long run only by taking the correct proportion between chaotic–ergodic and ordered trajectories (and the correct distribution of ordered trajectories).

The results of our present work provide a more complete view of order and chaos in BQM. Thus, they can be used as a tool in dealing with the most important question, which is the role of order and chaos in the derivation of the mean values of quantum observables in BQM.

## Data Availability

The datasets generated during the current study are available from the corresponding author upon reasonable request.

## Appendix A. Detection of Order and Chaos

Regarding the characterization of a Bohmian trajectory as ordered or chaotic, the numerical calculation of LCN is a demanding and time-consuming computational process. Thus, it is not affordable for the identification of order and chaos in large ensembles of trajectories—something necessary in the study of Born’s rule in BQM.

However, the ergodicity of the chaotic trajectories in our previous cases, allows us to avoid the calculation of the LCN of thousands of trajectories.

Namely, by making the colorplots of many chaotic trajectories initiated in various regions of the support of the wavefunction (the region of the x−y plane where ${\left|\Psi \right|}^{2}$ is not negligible), and finding whether they are locally or fully ergodic, we measured the size of their colorplots in the x−y plane. These sizes were almost identical due to their ergodic nature. On the other hand, the size of the colorplot of every ordered trajectory is always confined and cannot cover the size of the chaotic colorplots. Then, we integrated all the trajectories in our ensembles for long times and checked whether the size of their colorplots reached or not that of a single ergodic trajectory. If the answer was positive/negative, the trajectory was characterized as chaotic/ordered. This simple method depends significantly on the integration time since a chaotic trajectory can spend a lot of time in a certain region of space and behave like an ordered trajectory during that time. In our calculations, we worked with $t_{int}=3000$, which was found to be sufficiently large to jump from a confined region of space to a larger space and be considered chaotic by our algorithm. All our numerical calculations for this purpose were carried out in Python 3 by use of the LSODA adaptive integrator and with absolute error tolerance $a_{tol}=10^{-8}$ and relative error tolerance $r_{tol}=10^{-6}$ [51].
